# Global trends and cross-country inequalities in laryngeal cancer: A systematic analysis of the 2021 Global Burden of Disease study represented by China

**DOI:** 10.18332/tid/205796

**Published:** 2025-07-26

**Authors:** Zhizhen He, Yifan Hu, Xiuping Yang, Baoai Han, Shuang Li, Shuo Huang, Xiong Chen

**Affiliations:** 1 Department of Otorhinolaryngology, Head and Neck Surgery, Zhongnan Hospital of Wuhan University, Wuhan, China; 2 Sleep Medicine Centre, Zhongnan Hospital of Wuhan University, Wuhan, China

**Keywords:** laryngeal cancer, disease burden, risk factors, public health

## Abstract

**INTRODUCTION:**

Laryngeal cancer is one of the most common malignant tumors in the upper respiratory tract, accounting for approximately 4.5% of all malignant tumors. Smoking, drinking alcohol and occupational exposure are its main risk factors. Based on the Global Burden of Disease (GBD) database from 1990 to 2021, this study conducted a comparative analysis of laryngeal cancer-related data in China and around the world.

**METHODS:**

This is a secondary dataset analysis based on the Global Burden of Disease Study 2021. Chinese and global laryngeal cancer data for the period from 1990 to 2021 were extracted from the GBD database, and the Joinpoint regression model was used to analyze age-standardized incidence, prevalence, mortality, and DALYs. The inequality slope index (SII) and concentration index were calculated to assess health inequalities and risk factor attribution analysis was performed. Finally, a Bayesian hierarchical modeling method was used to predict the laryngeal cancer burden from 2022 to 2050.

**RESULTS:**

From 1990 to 2021, the ASPR in China increased from 7.83 per 100000 people to 9.86, while the ASIR in the world decreased from 15.27 per 100000 people to 12.56, showing an opposite trend. The global inequality index (SII) shows a downward trend but is still greater than 0 (SII was 3.70 in 2021), and the concentration index has changed from -0.23 to -0.13, indicating that health problems related to laryngeal cancer are concentrated in poor countries, but the inequality between poor and rich countries is narrowing. Among women in high-income countries, the attributed deaths caused by tobacco and alcohol consumption are 58.5% and 9.8%, respectively, which are much higher than the global average of 30.9% and 3%. The attributable proportions of DALYs caused by tobacco and alcohol consumption were 61.5% and 10.7%, respectively, which were much higher than the global average proportions of 29% and 3.1%. It is estimated that by 2050, the global incidence rate of laryngeal cancer will decrease to 2.020 per 100000 people, the mortality rate will decrease to 1.028 per 100000 people, the cancer incidence rate among Chinese men will be 0.453 per 100000 people, and the ASMR will decrease to 0.173 per 100000 people.

**CONCLUSIONS:**

The global burden of laryngeal cancer decreased overall from 1990 to 2021, but the incidence and prevalence in China are complex and may be influenced by urbanization and lifestyle changes. The incidence of laryngeal cancer in Chinese women continues to rise, which is worthy of attention. Low-income countries face greater challenges, where digital health technologies can help with early screening and treatment.

## INTRODUCTION

Laryngeal cancer is the second most common head and neck cancer, involving multiple anatomic regions, including the mouth, oropharynx, nasopharynx, hypopharynx, larynx, sinuses and salivary glands^1^, and is an important player in otorhinolaryngology. In 2020, 184615 newly diagnosed cases of LC and 99840 laryngeal cancer-related deaths were reported globally^2^. The pathogenesis of laryngeal cancer involves several risk factors. The most important of these are tobacco and alcohol consumption^3^. Tobacco use has been shown to be linearly associated with the development of throat cancer, with smokers having a 10 to 15 times higher risk than non-smokers and the heaviest smokers having a 30 times higher risk^4^. Exposure to several other environmental factors is also thought to potentially increase the risk of laryngeal squamous cell carcinoma, such as asbestos^5^. In addition, there are racial differences in the development of throat cancer, and studies have shown that African Americans develop the disease at a younger age and have higher incidence and mortality rates than Caucasians^6^. Over the past 40 years, while the overall incidence is declining, the 5-year survival rate for throat cancer has unfortunately fallen from 66% to 63%^7^, one of the few diseases where 5-year survival rates have decreased as medical technology has improved. The early symptoms of laryngeal cancer are insidious and often advanced when detected, and relevant data show that about 50% of patients present with advanced (stage III or IV) disease at diagnosis^8^, and the risk of death from LC in patients with advanced tumors is almost three times that of patients with initial tumors (stage I and II)^9^. This may be one of the reasons why the 5-year survival rate for laryngeal cancer continues to decline. Therefore, early screening and timely diagnosis are crucial to improve the cure rate and prognosis.

The Global Burden of Disease (GBD) study is a large-scale observational epidemiological project with global coverage that systematically records 369 disease indicators, including incidence, prevalence, mortality, and disability-adjusted life year rates^10^. A comprehensive study and analysis of the burden of laryngeal cancer in different regions and countries through these data is essential to develop more targeted prevention and control strategies. In order to better evaluate the influencing factors of laryngeal cancer burden, we selected relevant data of laryngeal cancer in China and around the world for analysis and comparison. China is a big tobacco country. Although it is a developing country, it has been in a stage of rapid development in the past 20 years, and its medical and health level has experienced a stage from weak to strong^11^, but there are some economic characteristics of unbalanced development^12^, which is a good subject for research on factors affecting the burden of laryngeal cancer.

This study utilized GBD data to conduct a novel comparative analysis of the burden data of laryngeal cancer in China and globally from 1990 to 2021. The study objective was to analyze the impact of age and sex on LC incidence, prevalence, mortality and DALYs rate using GBD 2021 data.

## METHODS

### Data source

This is a secondary dataset analysis based on the Global Burden of Disease Study 2021, which comprehensively evaluates the health losses caused by 371 diseases and injuries, as well as 288 causes of death, in 204 countries and regions using the latest epidemiological data and improved standardized methods. The estimation process of GBD is based on identifying multiple relevant data sources, including population censuses, household surveys, civil registration and vital statistics, disease registration, health service utilization, and other sources. The LC diagnostic codes ICD-10 used in this study were C32-C32.9, D02.0, D14.1, and D38.0, and ICD-9 were 161-161.9, 212.1, 231.0, and 235.6. A global health data exchange query tool was used to obtain the incidence rate, prevalence, mortality and DALYs of laryngeal cancer by age, gender, region, country and SDI from 1990 to 2021, including estimated values and 95% uncertainty interval. As we used publicly available data, ethical approval was not required.

### Joinpoint analysis

We used Joinpoint software to calculate the annual percent change (APC), average annual percent change (AAPC) and corresponding 95% confidence intervals (95% CI) for the incidence, prevalence, deaths, and DALYs rates of LC in China and globally from 1990 to 2021. Best-fitting models were obtained using the Monte Carlo permutation test, with a maximum of 5 connection points. Meanwhile, we used the Bayesian Information Criterion (BIC) to compare the goodness of fit of different models calculated by the jointpoint software and selected the model with the smallest criterion value for comparison and assessed the trends in disease burden. If the 95% CI for the AAPC estimate is greater than 0, it indicates an increasing trend; if less than 0, a decreasing trend; and if equal to 0, a stable trend^13^.

### SDI

The sociodemographic index (SDI) is a composite measure that combines income, education level, and fertility rates to assess the sociodemographic development and fertility status of a country or region. Based on SDI scores, the 204 countries and regions were classified into five categories: low (<0.47), low-middle (0.47–0.62), middle (0.63–0.71), high-middle (0.72–0.81), and high (>0.81).

### Incidence-to-mortality ratio

An alteration in the incidence-to-mortality ratio (IMR) may be employed as a means of elucidating potential concerns pertaining to overdiagnosis. An increase in the IMR over time, when the mortality rate remains stable or declines slowly, may indicate an increase in overdiagnosis. Conversely, should the mortality rate also demonstrate an increase, this would suggest that overdiagnosis is not the primary issue.

### Measurement health inequalities

Total incidence and age-age standardized incidence rates were extracted for inequality analysis. As per the recommendations of the World Health Organization, two standard measures, namely slope index of inequality (SII) and concentration index, were used to assess both absolute and relative income-related inequalities between countries. The SII represents the slope of the regression line that correlates the country-level age standardized incidence rate (ASIR) associated with LC with the weighted ranking of each country. The concentration index, a numerical integration of the area under the curve, ranging from -1 to 1, was used to assess the relative disparity in the burden of LC among countries, employing a Lorenz concentration curve based on cumulative incidence and cumulative population.

### Attribution analysis

To explore the relationship between laryngeal cancer and the main risk factors, we selected three main risk factors, tobacco, high alcohol use and occupational risks for analysis. In this study, for the risk analysis of tobacco use, based on the classification of the GBD database, the general item ‘tobacco’ was uniformly adopted for processing, covering the combined effects of ‘smoking’, ‘secondhand smoke’, and ‘chewing tobacco’, in order to reflect the relationship between overall tobacco-related exposure and the burden of laryngeal cancer.

### Predictive analysis

To predict the number of new cases and the incidence rate worldwide from 2022 to 2050, we used the INLA and BAPC packages to predict the global burden of disease through a Bayesian hierarchical model. The INLA package is used for efficiently processing complex spatio-temporal data, while the BAPC package focuses on Bayesian analysis of demographic data and is capable of dynamically integrating age, period and queue effects, making it suitable for long-term trend modeling^14^. Compared with traditional time series prediction methods (such as the ARIMA model), BAPC can not only better capture population structure changes and queue effects, but also has stronger adaptability and explanatory power in dealing with non-stationary data and complex long-term trends. The data used is from the Global Health Burden Database and has been standardized and preprocessed. Furthermore, we conducted model residual diagnosis based on the INLA framework, evaluated the normality of the residuals using Shapiro-Wilk tests, and evaluated the model fitting effect in combination with the bias information of the model (such as DIC).

### Statistical analysis

This study analyzed the incidence, prevalence, mortality, DALYs and age-standardized rates (ASRs) at global, regional, and country levels. The estimated annual percentage changes (EAPCs) were also calculated to assess trends in these indicators. The calculation of EAPCs is based on the linear regression of natural logarithm [ln (rate)]^15^. Moreover, the long-term trend of ASR has been examined over the past 32 years. If the lower limit of the 95% confidence interval (CI) of EAPCs is greater than 0, then an upward trend can be observed. If the upper limit is less than 0, then a downward trend can be observed. This analytical method helps us understand the temporal dynamics of the incidence of LC. The strength of Spearman’s correlation (ρ) was interpreted based on its absolute value: |ρ| <0.3 was considered weak, 0.3≤ |ρ| <0.5 moderate, and |ρ| ≥0.5 strong, as applied in previous validation studies^16^. For pairwise comparisons across SDI regions and sexes, the false discovery rate (FDR) correction was applied to adjust for multiple comparisons and reduce the risk of type I error. All data analyses were completed in the R software version 4.3.2, with a statistical significance level set at α=0.05.

## RESULTS

### Description of LC burden in China and globally

From 1990 to 2021, the age standardized incidence rate (ASIR) and age-standardized prevalence rate (ASPR) of LC showed a downward trend globally. For ASIR, the global ASPR declined from 15.27 (95% UI: 14.54–16.06) to 12.56 (95% UI: 11.76–13.49) per 100000 people. The situation in China is quite different, with almost no change in ASIR, from 1.82 per 100000 people (95% UI: 1.5–2.13) to 1.79 (95% UI: 1.4-2.26). ASPR has even increased, from 7.83 per 100000 people (95% UI: 6.47–9.13) to 9.86 (95% UI: 7.81–12.35). Consistently, the age standardized mortality rate (ASMR) and age standardized DALYs rate (ASDR) of LC have shown significant declines both in China and globally. The ASMR in China has decreased from 1.59 per 100000 people (95% UI: 1.32–1.86) to 0.94 (95% UI: 0.74–1.17), while the global ASMR has decreased from 2.15 per 100000 people (95% UI: 2.01–2.28) to 1.35 (95% UI: 1.26–1.45). The ASDR in China has decreased from 40.37 (95% UI: 33.13–47.6) per 100000 people to 22.73 (95% UI: 17.67–28.65), while globally it has decreased from 59.3 (95% UI: 55.47–63.1) per 100000 people to 35.8 (95% UI: 33.29–38.54). In terms of annual average rate of change (EAPC), from 1990 to 2021, the EAPCs for ASIR, ASPR, ASMR, and ASDR in China and globally were 0.04 (95% UI: -0.11–0.2), 0.9 (95% UI: 0.73–1.07), -1.74 (95% UI: -1.83 – -1.65), -1.91 (95% UI: -2.01 – -1.81), -1.09 (95% UI: -1.17 – -1.01), -0.76 (95% UI: -0.83 – -0.7), -1.66 (95% UI: –1.74 – -1.58), and -1.82 (95% UI: -1.9 – -1.73), respectively. In contrast, there are some differences in the burden of laryngeal cancer between China and the world, mainly reflected in the incidence rate and prevalence rate, while there is no significant difference in mortality and DALYs (Table 1). The changing trend of female LC burden is roughly consistent with the changing trend of the total population (Table 2). It is worth noting that from 1990 to 2021, the proportion of male laryngeal cancer patients worldwide slightly decreased in various indicators, while the proportion of male laryngeal cancer patients in China slightly increased. Whether in 1990 or 2021, males accounted for over 80% of the total incidence of laryngeal cancer, and the trend of changes in male laryngeal cancer burden can to some extent reflect the trend of the total population (Table 3). In addition, compared to 1990, the IMR of LC in China and globally has increased in 2021 (Table 4).

**Table 1:** The number of LC cases across all age groups, age-standardized incidence rate, prevalence rate, mortality rate and DALYs rate and corresponding EAPC in the total population of China and globally in 1990 and 2021

*Location*	*Measure*	*1990*		*2021*		*1990-2021 EAPC*
		*All-ages cases*	*Age-standardized* *rate per 100000* *people*	*All-ages cases*	*Age-standardized* *rate per 100000* *people*	
		*n (95% UI)*	*n (95% UI)*	*n (95% UI)*	*n (95% UI)*	*% (95% CI)*
China	Deaths	12870 (10565–15143)	1.59 (1.32–1.86)	19799 (15580–25023)	0.94 (0.74–1.17)	-1.74 (-1.83 – -1.65)
China	DALYs	362503 (295796–428646)	40.37 (33.13–47.6)	493848 (382572–626010)	22.73 (17.67–28.65)	–1.91 (-2.01 – -1.81)
China	Prevalence	69309 (56887–81340)	7.83 (6.47–9.13)	217849 (171469–273851)	9.86 (7.81–12.35)	0.9 (0.73–1.07)
China	Incidence	15434 (12624–18174)	1.82 (1.5–2.13)	38905 (30370–49486)	1.79 (1.4–2.26)	0.04 (-0.11–0.2)
Global	Deaths	85790 (80409–91208)	2.15 (2.01–2.28)	117252 (109355–125952)	1.35 (1.26–1.45)	-1.66 (-1.74 – -1.58)
Global	DALYs	2475842 (2313814–2636993)	59.3 (55.47–63.1)	3143309 (2922792–3383514)	35.8 (33.29–38.54)	-1.82 (-1.9 – -1.73)
Global	Prevalence	628532 (598814–660379)	15.27 (14.54–16.06)	1103684 (1033145–1186560)	12.56 (11.76–13.49)	-0.76 (-0.83 – -0.7)
Global	Incidence	125175 (118981–131639)	3.07 (2.92–3.23)	200883 (186941–216098)	2.29 (2.13–2.47)	-1.09 (-1.17 – -1.01)

LC: laryngeal cancer. DALYs: disability-adjusted life years. EAPC: estimated annual percentage change.

**Table 2:** The number of LC cases across all age groups, age-standardized incidence rate, prevalence rate, mortality rate and DALYs rate and corresponding EAPC in females in China and globally in 1990 and 2021

*Location*	*Measure*	*1990*		*2021*		*1990–2021 EAPC*
		*All-ages cases*	*Age-standardized* *rate per 100000* *people*	*All-ages cases*	*Age-standardized* *rate per 100000* *people*	
		*n (95% UI)*	*n (95% UI)*	*n (95% UI)*	*n (95% UI)*	*% (95% CI)*
China	Deaths	2298 (1314–2947)	0.56 (0.33–0.71)	3338 (1977–4603)	0.3 (0.18–0.42)	-2.13 (-2.23 – -2.03)
China	DALYs	61224 (32586–78704)	13.66 (7.52–17.45)	77081 (46252–106174)	7.01 (4.19–9.66)	-2.32 (-2.42 – -2.22)
China	Prevalence	11951 (6923–15218)	2.7 (1.62–3.41)	36539 (23494–49819)	3.27 (2.11–4.44)	0.69 (0.56–0.83)
China	Incidence	2671 (1519–3422)	0.63 (0.37–0.8)	6394 (3873–8782)	0.58 (0.35–0.79)	-0.27 (-0.41 – -0.14)
Global	Deaths	10528 (8183–11773)	0.49 (0.39–0.55)	16859 (14209–19876)	0.37 (0.31–0.43)	-1.12 (-1.21 – -1.04)
Global	DALYs	294696 (220024–332508)	13.49 (10.13–15.19)	449130 (377885–540000)	9.93 (8.33–11.93)	-1.15 (-1.23 – -1.07)
Global	Prevalence	79161 (67109–85641)	3.67 (3.12–3.96)	163759 (144050–186895)	3.57 (3.14–4.09)	-0.22 (-0.28 – -0.16)
Global	Incidence	15297 (12506–16734)	0.71 (0.58–0.78)	29094 (24975–33680)	0.63 (0.54–0.73)	-0.52 (-0.59 – -0.45)

LC: laryngeal cancer. DALYs: disability-adjusted life years. EAPC: estimated annual percentage change.

**Table 3:** The number of LC cases across all age groups, age-standardized incidence rate, prevalence rate, mortality rate and DALYs rate and corresponding EAPC in males in China and globally in 1990 and 2021

*Location*	*Measure*	*1990*		*2021*		*1990–2021 EAPC*
		*All-ages cases*	*Age-standardized* *rate per 100000* *people*	*All-ages cases*	*Age-standardized* *rate per 100000* *people*	
		*n (95% UI)*	*n (95% UI)*	*n (95% UI)*	*n (95% UI)*	*% (95% CI)*
China	Deaths	10571 (8433–12693)	2.81 (2.29–3.35)	16462 (12222–21222)	1.68 (1.27–2.13)	-1.68 (-1.77 – -1.58)
China	DALYs	301279 (237979–364048)	68.62 (54.7–82.4)	416766 (304821–544631)	39.58 (29.17–51.44)	-1.8 (-1.91 – -1.69)
China	Prevalence	57358 (46014–68664)	13.31 (10.74–15.77)	181310 (133834–234748)	16.86 (12.58–21.67)	0.94 (0.75–1.12)
China	Incidence	12763 (10155–15390)	3.16 (2.56–3.79)	32510 (24069–42391)	3.12 (2.34–4.04)	0.08 (-0.08–0.24)
Global	Deaths	75262 (70749–80447)	4.12 (3.87–4.4)	100393 (93351–108830)	2.49 (2.31–2.69)	-1.79 (-1.87 – -1.71)
Global	DALYs	2181146 (2048786–2333042)	110.04 (103.4–117.38)	2694179 (2491890–2926037)	64.13 (59.35–69.57)	-1.93 (-2.02 – -1.84)
Global	Prevalence	549372 (522107–579828)	28.43 (27–29.99)	939924 (876345–1011203)	22.54 (21.03–24.21)	-0.88 (-0.94 – -0.81)
Global	Incidence	109879 (104446–116063)	5.8 (5.5–6.12)	171789 (159470–186042)	4.16 (3.86–4.5)	-1.21 (-1.29 – -1.13)

LC: laryngeal cancer. DALYs: disability-adjusted life years. EAPC: estimated annual percentage change.

**Table 4:** IMR of laryngeal cancer in China and globally in 1990 and 2021

*Location*	*Sex*	*IMR (95% CI)*	
		*1990*	*2021*
China	Both	1.199 (0.834–1.72)	1.965 (1.214–3.176)
	Female	1.162 (0.515–2.605)	1.916 (0.841–4.442)
	Male	1.207 (0.8–1.825)	1.975 (1.134–3.469)
Global	Both	1.459 (1.305–1.637)	1.713 (1.484–1.976)
	Female	1.453 (1.062–2.045)	1.726 (1.257–2.37)
	Male	1.46 (1.298–1.64)	1.711 (1.465–1.993)

IMR: incidence-to-mortality ratio.

**Figure 1:**
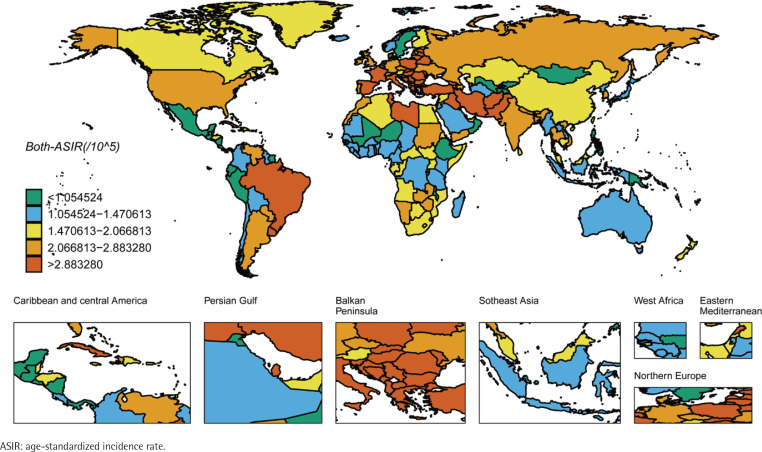
ASIR in the total population for laryngeal cancer across 204 countries and regions worldwide in 2021

**Figure 2:**
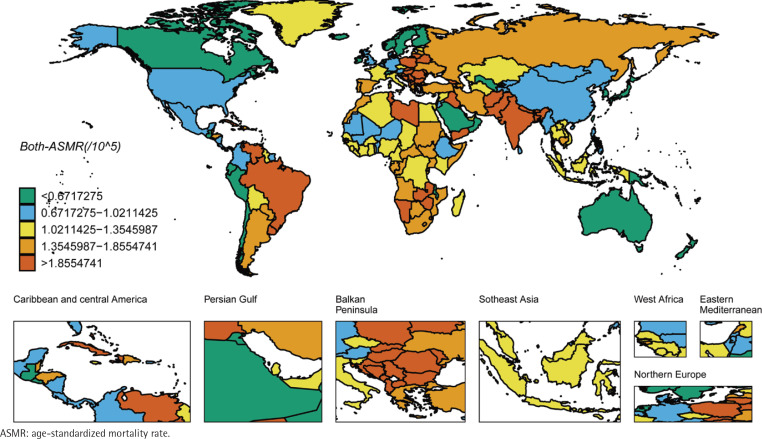
ASMR in the total population for laryngeal cancer across 204 countries and regions worldwide in 2021

**Figure 3:**
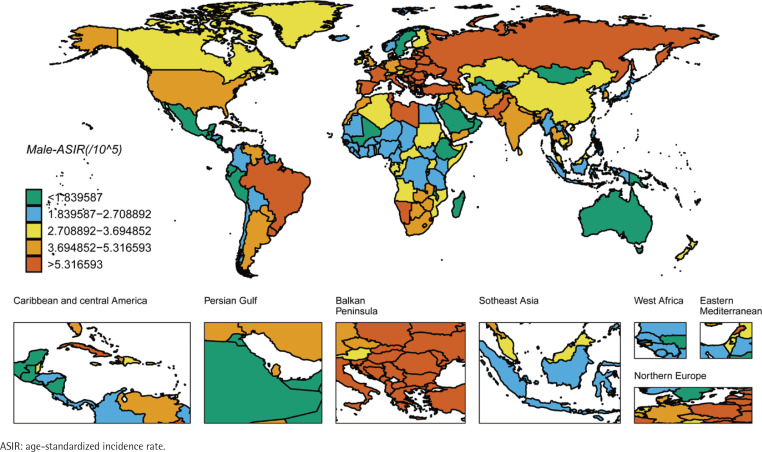
ASIR in males for laryngeal cancer across 204 countries and regions worldwide in 2021

**Figure 4:**
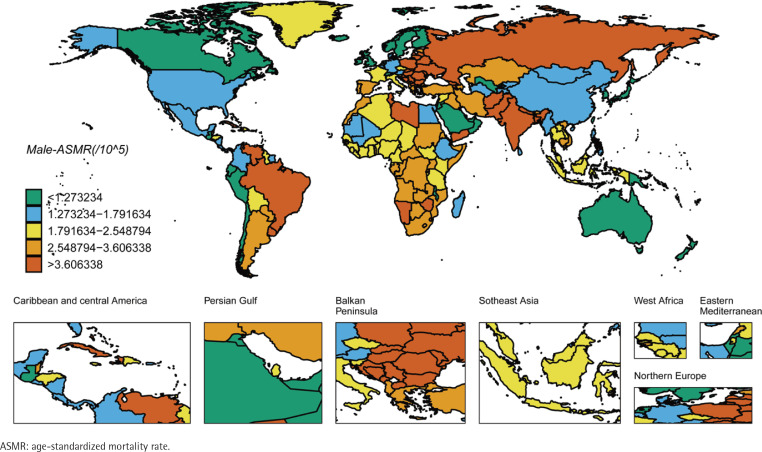
ASMR in the males for laryngeal cancer across 204 countries and regions worldwide in 2021

**Figure 5:**
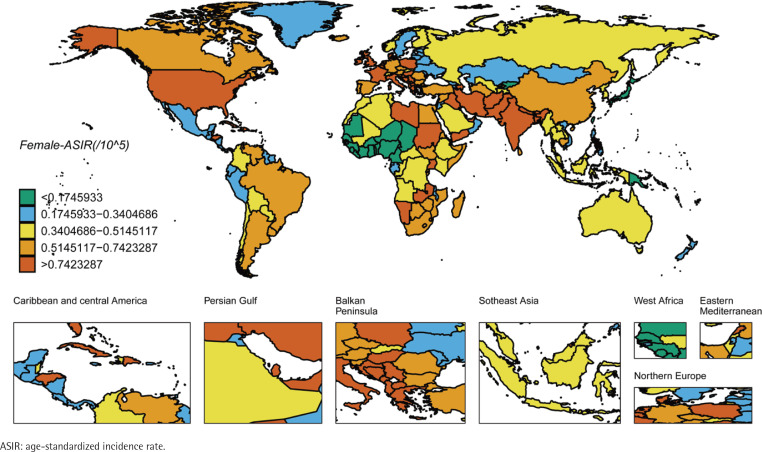
ASMR in the females for laryngeal cancer across 204 countries and regions worldwide in 2021

**Figure 6:**
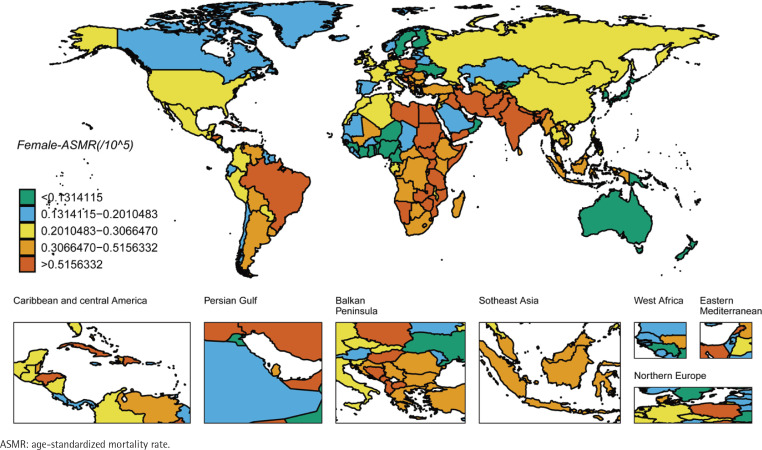
ASMR in the females for laryngeal cancer across 204 countries and regions worldwide in 2021

We ranked the ASIR and ASMR of different countries and regions, and drew six world maps with total population, male and female research subjects respectively. Firstly, the regions with high global ASIR and ASMR are concentrated in South America, Europe and western Asia (Figures 1 and 2). China’s ASIR and ASMR are at a relatively low level globally. If only men are considered, the regions with high global ASIR and ASMR are concentrated in South America, Europe and Russia. The ASIR and ASMR of Chinese men are still at a relatively low level globally (Figures 3 and 4). If only women are considered, the regions with high levels of ASIR and ASMR globally are concentrated in South America, India, the United States and other areas, while the ASMR and ASIR of Chinese women are at a relatively high level globally (Figures 5 and 6).

We have also analyzed the incidence rate, incidence, mortality and DALYs of LC in different age groups in China and the world in 1990 and 2021. The incidence and prevalence rate of LC in China and the world, as well as the corresponding number of cases, are rising first and then declining with the increase of age. Compared to 1990, there is a significant increase in the prevalence of all age groups in China in 2021, and the incidence is nearly flat. However, the incidence rate and prevalence rate of all age groups in the world showed a downward trend. In 2021, the age of onset in China and globally is mainly concentrated in the ages 45–84 years, with a peak in the number of cases occurring at 65–69 years. The highest incidence rate is in the age group 70–74 years in China, while globally it is in the age group of 65–69 years (Supplementary file: Figures 1A–1B and 1E–1F). From 1990 to 2021, LC mortality and disability-adjusted life decreased significantly annually in all age groups in China and globally. Deaths in all age groups in China and globally are mainly concentrated in the age group of 50–89 years. The age group with the highest number of deaths is 65–69 years, and the age group with the highest mortality rate is 85–89 years (Supplementary file: Figures 1C–1D and 1G–1H).

We illustrate the correlation between ASIR, ASMR, and SDI. With the increase of SDI, the total population and ASIR of males show a trend of first increasing, then decreasing, and then increasing again (Supplementary file: Figures 2A and 2C). The ASIR of women remains stable (Supplementary file: Figure 2E). In addition, the total population and ASDR of males showed a trend of first increasing and then decreasing with the increase of SDI (Supplementary file: Figures 2B and 2D). The ASDR of women fluctuates with the increase of SDI, but overall, it shows a downward trend (Supplementary file: Figure 2F). It is worth noting that the ASIR and ASMR of the total population and males in China are lower than expected, while the ASIR and ASMR of Chinese females are very close to expected levels.

### Joinpoint analysis of LC burden between China and the world

Since 1990, the ASIR and ASPR of LC in China’s total population have decreased first and then increased. The decline of ASIR was concentrated in 1990–1999, the APC was -1.16%, and the APC increased significantly from 2007 to 2011, when the APC was 1.97%. The ASPR showed an even greater increase, especially from 2007 to 2012, when the APC was 2.40%. ASMR and ASDR showed a downward trend in general, and both showed the fastest decline from 2004 to 2007, with APC of -3.62% and -3.58%, respectively (Supplementary file: Figures 3A–3D). ASIR, ASPR, ASMR and ASDR of the global total population all showed a downward trend, and the fastest declining periods were 1995–2002, 1996–2002, 1994–2007 and 1994–2007, with APC of -1.94%, -1.75% and -2.17%, -2.35%, respectively (Supplementary file: Figures 3E–3H).

For males, due to the high proportion of laryngeal cancer cases, the speed of changes in various indicators and corresponding years are consistent with the total population. The ASIR and ASPR of Chinese male LC showed a trend of first decreasing and then rapidly increasing. The main years of increase were from 2007 to 2012, with APCs of 1.87% and 2.57% (Supplementary file: Figures 4A–4B), respectively. ASMR and ASDR showed an overall downward trend, both experiencing the fastest decline from 2004 to 2007, with APCs of -3.67% and -3.57% (Supplementary file: Figures 4C–4D). The ASIR, ASPR, ASMR, and ASDR of males worldwide are all showing a downward trend. The fastest decline periods were from 1995 to 2002, 1996 to 2002, 1994 to 2007, and 1994 to 2007, with APCs of -2.08%, - 1.86%, - 2.30%, - 2.51% (Supplementary file: Figures 4E–4H).

From 1990 to 2021, the ASIR of Chinese women’s LC fluctuated significantly, showing a clear upward trend from 2015 to 2021, with an APC of 1.37% (Supplementary file: Figure 5A). ASPR shows a continuous upward trend, with the fastest increase from 2015 to 2021, and APC at 1.91% (Supplementary file: Figure 5B). ASMR and ASDR showed a downward trend (Supplementary file: Figures 5C–5D). However, the ASIR and ASPR of women worldwide have gradually decreased to a stable level, with ASIR having an APC of 0.03% between 2013 and 2021, and ASPR having an APC of 0 between 2004 and 2021. In addition, both Chinese and global female ASMR and ASDR are showing a downward trend (Supplementary file: Figures 5E–5H).

There are certain differences in the trends of ASIR and ASPR between China and global, both in terms of total population and male or female. It is worth noting that the ASIR and ASPR of Chinese male LC have shown an upward trend in the past decade, while the ASIR and ASPR of Chinese female LC have shown an upward trend in the past six years, which is opposite to the global trend of ASIR and ASPR of LC. This abnormal trend deserves more attention.

### Analysis of health inequality

The interaction effect of SDI ranking and gender on laryngeal cancer incidence in 1990 and 2021 reveals a clear disparity (p<0.001) (Supplementary file: Figure 6A). Incidence in men increases steeply with higher SDI, while remaining relatively stable in women. Concentration curves for 1990 and 2021 show that the slope of male inequality rose from 4.22 to 6.14, indicating worsening disparity, whereas female inequality remained consistently low (0.58–0.76), reflecting notable gender differences (Supplementary file: Figure 6B). The male curve deviates more from the diagonal than the female curve, suggesting greater inequality in disease burden among men across SDI levels (Supplementary file: Figure 6C). Over time, the SII value for males increased steadily, while that for females remained stable, with linear regression confirming a growing trend of male inequality and minimal change among females (Supplementary file: Figure 6D).

### Attribution analysis

We selected three main risk factors associated with laryngeal cancer (smoking, high alcohol use, and occupational risk) and explored the differences in these factors globally, in China, and in countries with different SDI levels. Data show that the global mortality rate caused by smoking is 66.5%, while in China it is 77%. The global proportion of deaths caused by high alcohol use is 12.4%, with 14.5% in China. The mortality rate of occupational risk is 5.9% globally and 5.2% in China (Supplementary file: Figure 7A). In the male population, smoking accounts for 72.4% of global deaths and 86.3% in China. The proportion of high alcohol use is 14% globally and 17.2% in China (Supplementary file: Figure 7B). Among the female population, smoking accounts for 30.9% of deaths globally and 31.5% in China. The global proportion of high alcohol use among women is 3%, while in China it is 1.5% (Supplementary file: Figure 7C). These differences indicate that China has a heavier burden on smoking and high alcohol use compared to the global level, and this difference is more pronounced among the male population. The impact of smoking and high alcohol use on laryngeal cancer intensifies with the improvement of economic development level in countries with different SDI levels. The proportion of deaths caused by smoking in high SDI countries is 76.2%, high alcohol use is 17.4%, while in low SDI countries it is 47.7% and 6.9%, respectively. The impact of occupational risk is 6% in high SDI countries and 4% in low SDI countries (Supplementary file: Figure 7A). This indicates that in high-income countries, the prevalence of smoking and high alcohol use is higher, leading to a more significant risk of throat cancer. Low-income countries, on the other hand, face greater occupational risk issues, which may be related to their poorer working environments and safety standards.

Global data show that the proportion of DALYs caused by smoking in laryngeal cancer worldwide is 65.6%, while in China it is 77.1%. The global proportion of high alcohol use is 13%, with China accounting for 15.8% (Supplementary file: Figure 8A). In the male population, the DALYs of laryngeal cancer caused by smoking are 71.6% globally and 86% in China (Supplementary file: Figure 8B); Among the female population, the DALYs of laryngeal cancer caused by smoking are 29% globally and 29.6% in China (Supplementary file: Figure 8C). In countries with different SDI levels, the impact of smoking and high alcohol use on laryngeal cancer increases with the level of economic development. Especially in high SDI countries, smoking related laryngeal cancer DALYs account for 73.6%, high alcohol use accounts for 20.4%, while in low SDI countries they are 45.8% and 7.3% (Supplementary file: Figure 8A). This indicates that countries with higher levels of economic development face a more severe risk of throat cancer due to the prevalence of smoking and alcohol use.

In terms of the mortality rate attributable to smoking, men worldwide have consistently been significantly higher than women. In 1990, the proportion of men was approximately 3.3 per 100000. Although it slightly decreased in 2021, it was still higher than 3.0 per 100000. For women, it has remained consistently below 0.4 per 100000, with little change. The mortality rate caused by high alcohol use also shows gender differences. Between 1990 and 2021, the mortality rate for men decreased from approximately 0.6 per 100000 to 0.5 per 100000, and for women, it decreased from 0.08 per 100000 to 0.05 per 100000. The mortality rate caused by occupational exposure is generally low, but it is always higher in men than in women. Although the difference is small, it still has certain significance (Supplementary file: Figure 9A). Relatively speaking, the gender differences in China are more significant. The mortality rate attributable to smoking among Chinese men was approximately 3.1 per 100000 in 1990 and rose to 3.4 per 100000 in 2021, significantly higher than that among women (<0.2 per 100000), with a gap of more than 15 times. The mortality rate caused by high alcohol use is also relatively high. In 2021, it was approximately 0.7 per 100000 for men, and it slightly increased after 2010. Women are still at an extremely low level (<0.1 per 100000). In terms of occupational exposure, the proportion of men decreased from 0.3 per 100000 in 1990 to 0.2 per 100000 in 2021, while that of women remained consistently below 0.05 per 100000 (Supplementary file: Figure 9B).

### Predicted trends

We used ASIR and ASMD from 1990 to 2021 to predict the China and global trend of laryngeal cancer from 2022 to 2050. From 2021 to 2050, the global total population, as well as the ASIR of males and females, are expected to continue to decline, with ASIR decreasing from 2.294 per 100000 people, 4.162 per 100000 people, and 0.634 per 100000 people to 2.020 per 100000 people, 3.526 per 100000 people, and 0.622 per 100000 people (Supplementary file: Figure 10B). The ASMR of the global population, male and female, is expected to continue to decline, from 1.35 per 100000 people, 2.491 per 100000 people, 0.367 per 100000 people to 1.028 per 100000 people, 1.797 per 100000 people, 0.325 per 100000 people, respectively (Supplementary file: Figure 10D). In contrast, the situation in China is not very optimistic. In 2021–2033, the ASIR of Chinese LC men is expected to increase from 0.789 per 100000 people to 0.829 per 100000 people, and the incidence rate will reach the peak. From 2033 to 2050, the ASIR of males will continue to decline to 0.777 per 100000 people. In 2037, the incidence rate of the total LC population in China will reach the peak, and the ASIR will reach 0.477 per 100000 people. From 2037 to 2050, the ASIR of the total LC population in China will continue to decline to 0.453 per 100000 people. From 2021 to 2050, the ASIR of Chinese women will continue to rise, increasing from 0.139 per 100000 people to 0.186 per 100000 people (Supplementary file: Figure 10A). Fortunately, the ASMR of both males and females in China’s total population is expected to continue to decline. It is expected that by 2050, the ASMR of the total population will decrease from 0.229 per 100000 people to 0.173 per 100000 people. The ASMR of males will decrease from 0.412 per 100000 people to 0.257 per 100000 people, while the ASMR of females will decrease from 0.072 per 100000 people to 0.063 per 100000 people (Supplementary file: Figure 10C). All projections were generated using the INLA-based BAPC model. Model diagnostics, including DIC values and Shapiro-Wilk normality tests for residuals, are provided (Supplementary file: Table 1).

## DISCUSSION

We assessed the global and Chinese throat cancer burden and its trends from 1990 to 2021, and analyzed risk factors associated with death and DALYs. To understand these differences more fully, we conducted an in-depth subgroup analysis based on SDI, gender, and age, highlighting China’s differences on these factors in particular. It is important to note that this study is the first comparative analysis of laryngeal cancer burden in China and globally based on 2021 data and has important public health implications.

From 1990 to 2021, global laryngeal cancer ASIR and ASPR showed a downward trend, while the situation in China was relatively complicated, with ASIR almost unchanged and ASPR slightly increasing. This might be related to the fact that China’s extensive development model has exacerbated environmental pollution^17^ and that the diet structure has shifted from a plant-based diet to a Western-style diet high in fat and animal foods^18^. Relevant studies show that animal products and cereals (OR=1.5; 95% CI: 1.1– 2.1) and fats (OR=1.8; 95% CI: 1.4–2.3) patterns were positively associated with laryngeal cancer risk whereas a linear inverse trend in laryngeal cancer risk was evident for the antioxidant vitamins and fiber pattern^19^. Furthermore, a study in Europe confirmed that indoor air pollution might play a role in head and neck tumors^20^. In contrast, ASMR and ASDR for laryngeal cancer have shown significant declines both in China and globally. This may be due to the emergence of new therapies, such as the application of programmed death-1 checkpoint inhibitors in immunotherapy, which has dramatically changed the treatment landscape for cancer patients^21^. In addition, the renewal of the concept of comprehensive therapy has also brought efficient treatment methods. It has been reported that for patients with advanced laryngeal cancer, adding immunotherapy to radical chemoradiotherapy is likely to improve long-term survival while minimizing the negative effects of treatment^22^. However, these changes may be more reflected in the survival rate of diagnosed cases, and the overall disease burden has not been significantly reduced.

Whether in 1990 or 2021, men accounted for more than 80% of the total incidence of laryngeal cancer, which further indicates that men are still the main population with a high incidence of laryngeal cancer. It is worth noting that from 1990 to 2021, the proportion of men with laryngeal cancer decreased globally, but the proportion of men with laryngeal cancer increased slightly in China. Relevant studies have shown that smoking is the main cause of throat cancer. Nearly half of adult men in China smoke. By the 2010s, smoking was responsible for about one-fifth of all adult male deaths, and that proportion is rising^23^. This may have contributed to an increased burden of throat cancer. In addition, ASIR and ASPR of Chinese women show a rapidly increasing trend from 2015 to 2021, and relevant studies show that the age of women drinking alcohol is getting younger and younger, and the drinking rate is getting higher and higher^24^. Although smoking and alcohol consumption remain major risk factors, there is evidence that women may be more susceptible to the harmful effects of these behaviors and are at relatively higher risk than men^25^.

Globally, in 2021, there was a general decline in incidence and prevalence across all age groups compared to 1990. In China, the prevalence has increased significantly across all age groups. This may be related to the uneven implementation of public health interventions specific to China in the early 21st century, insufficient medical resources in some places, and the large gap between urban and rural areas. Firstly, studies have shown that the efficiency values of urban and industrial pollution control in China in 1991 and 2019 are less than 0.2 and 0.5, respectively, indicating that urban and industrial pollution control is far from achieving results^26^. Secondly, a study of medical conditions in western China from 2011 to 2021 found that although public health services have improved overall, they are still uneven^27^. Finally, our study found that in 2021, the age of onset in China and globally is mainly concentrated in the age group 45–84 years, while deaths are mainly concentrated in the age group 50–89 years. The age group with the highest number of deaths was 65–69 years, likely due to an ageing population combined with increasing incidence placing a considerable burden on health and social care systems^28^.

In 1990 and 2021, SII analyses of incidence showed a positive association between age-standardized incidence and the SDI index. This phenomenon may reflect that economic development and the abundance of medical resources directly affect the diagnosis and treatment of laryngeal cancer. Furthermore, in low-income countries, there is a lack of funding for prevention services. And community health workers are often insufficient in number and lack of training^29^, which is reflected in the lack of means of examination, resulting in late case detection, low incidence, and high mortality due to inadequate medical conditions. In rich countries, early diagnosis and advanced treatment can help reduce the burden of disease. Declines in the concentration index in 1990 and 2021 indicate some progress in reducing inequality in the global laryngeal cancer burden, mostly in high- and middle-income countries with universal early diagnostic tests and advanced treatments. China’s SDI index has increased significantly since 1990, and its position is close to the diagonal, indicating that China has made contributions in the field of economic development and public health, which may be related to the in-depth promotion of China’s medical reform^30^ and the implementation of nutrition policies and healthy China strategies^31^.

We explored the differences among the three major risk factors associated with laryngeal cancer globally, in China, and in countries with different SDI grades. The proportion of deaths from smoking and high alcohol consumption in China is much higher than the global average, reflecting the prevalence of these behaviors in China and their significant impact on health. The study showed that smoking rates among rural men, especially those born after 1990, increased from 40.2 percent in 2007 to 52.1 percent in 2018^32^. In addition, China’s alcohol market has grown rapidly over the past 30 years and is now one of the largest in the world. The annual consumption of alcohol per person in China in 2016 was 7.2 L, but in 2005 it was only 4.1 L^33^. These are all factors contributing to the increasing burden of laryngeal cancer in China. Low-income countries, on the other hand, face more occupational risk challenges^34^, which may be closely related to poor working conditions and safety standards. Therefore, the difference in laryngeal cancer burden is not only related to the socioeconomic level and the distribution of medical resources, but also closely related to the lifestyle of the population, the working environment and health policies. At present, digitizing healthcare is a promising solution to alleviate the dilemma of high disease burden and low healthcare expenditure in low-income countries^35^. The rapid development of artificial intelligence software and quantum computing hardware will accelerate its realization^36^.

### Limitations

This study still has some limitations. First of all, although we used the standardized data provided by the GBD database, its basic data came from the National Health Survey, hospital records and the cancer registration system. There are significant differences in data quality and completeness among different regions, especially in low-income countries and areas with sparse data, where there may be risks of underreporting or misreporting, affecting the accuracy and representativeness of the estimation results. Secondly, although SDI is an important indicator for measuring the level of social and economic development, it cannot fully reflect the complex social factors that affect disease outcomes, such as the accessibility of medical services, cultural norms and the intensity of policy intervention. Furthermore, the current analysis fails to incorporate certain gender-specific variables, such as hormone levels, occupational exposure patterns, and reproductive factors etc., which to some extent limits our in-depth understanding of the mechanism of gender differences in laryngeal cancer. Since the GBD data are aggregation-level data and lack individual-level information on hormones, occupational categories and lifestyles, we are unable to directly consider these potential influencing factors in the model. However, existing studies have suggested that these variables may play a key role in the differences in the burden of laryngeal cancer between men and women. Future studies should explore them in depth in combination with higher resolution individual data. In terms of trend analysis, we adopted the EAPC method to estimate the long-term changes in morbidity and mortality. However, this method is based on the logarithmic assumption of the linear regression model and may not be able to fully capture the nonlinear trends caused by public health emergencies, technological innovations in treatment, or policy interventions. Furthermore, since the prediction model itself relies on historical data, its sensitivity to future changes is uncertain, especially in the context of rapid changes in smoking behavior, environmental exposure or intervention strategies, the stability of the prediction results may be affected. Furthermore, since most of the data are integrated at the national or regional level, some of the analyses in this study inevitably have ecological fallacy, that is, the correlations at the group level may not be inferred to the individual level. Although we have explored gender differences, in some cultural contexts, reports on women’s smoking and drinking may be systematically underestimated, thereby affecting the authenticity of gender comparison results. Meanwhile, the promotion and popularization of emerging treatment methods have not yet been incorporated into the prediction model of GBD, which may also affect the true evolution of disease outcomes.

## CONCLUSIONS

This study analyzes the burden and trends of laryngeal cancer in China and globally from 1990 to 2021 and reveals that the global burden of laryngeal cancer has decreased, but the changes in incidence and prevalence in China are more complex and may be related to urbanization, environmental pollution, lifestyle, and high-risk behaviors. Chinese male groups bear a heavy burden, despite the progress of tobacco control policies, but risk behaviors are still common, and further control needs to be strengthened. The ASIR and ASPR of LC in Chinese women have both increased in the past six years, and it is predicted that by 2050, the ASIR in women will continue to rise, which needs great attention. China’s SDI has improved significantly, but it is still unbalanced between regions and groups. In addition, the plight of laryngeal cancer burden faced by low-income countries may be alleviated in the future through the digitization of healthcare.

## Supplementary Material



## Data Availability

The data supporting this research are available from the following link: https://vizhub.healthdata.org/gbd Results/
