# Supplementary material for: Global trends and cross-country inequalities in laryngeal cancer: A systematic analysis of the 2021 Global Burden of Disease study represented by China
Source: Tob Induc Dis. 2025 Jul 26;23:10.18332/tid/205796. doi: 10.18332/tid/205796 (PMC12305555; doi:10.18332/tid/205796)
Supplement: Supplementary file 1 [file TID-23-107-s1.pdf]

Supplementary Materials

Supplementary file: Figure 1. Comparison of incidence rates, prevalence rates, mortality rates, and DALYs rates by age group and their crude rates from 1990 to 2021 (A-D: China; E-H: Globally). The The error bars and shaded area represent the 95% UI. DALYs, disability-adjusted life years; UI, uncertainty interval.

Supplementary file: Figure 2. Age-standardized burden rates of lung cancer across 204 countries and regions by Socio-demographic Index (SDI), 1990–2021. Each dot represents a country or region, and the burden rates include incidence (ASIR) and mortality (ASMR). The black line represents a smoothed trend fitted using LOESS regression across all observations. The ρ and p-values shown in each panel represent the Spearman correlation coefficient and its significance level between SDI and the respective burden rate.China is labeled for emphasis. (A–B) both sexes; (C–D) males; (E–F) females.ASIR: age-standardized incidence rate; ASMR: age-standardized mortality rate.

Supplementary file: Figure 3. The APC of ASIR, ASPR, ASMR, and ASDR in the total population of LC in China (A-D) and globally (E-H) from 1990 to 2021 (* indicates P-value < 0.05, statistically significant). (A, E) ASIR; (B, F) ASPR; (C, G) ASMR; (D, H) ASDR. APC, annual percent change**.**ASIR: age-standardized incidence rate; ASMR: age-standardized mortality rate.LC: laryngeal cancer

Supplementary file: Figure 4. The APC of ASIR, ASPR, ASMR, and ASDR in male LC in China (A-D) and globally (E-H) from 1990 to 2021 (* indicates P-value < 0.05, statistically significant). (A, E) ASIR; (B, F) ASPR; (C, G) ASMR; (D, H) ASDR**.**

Supplementary file: Figure 5. The APC of ASIR, ASPR, ASMR, and ASDR in female LC in China (A-D) and globally (E-H) from 1990 to 2021 (* indicates P-value < 0.05, statistically significant). (A, E) ASIR; (B, F) ASPR; (C, G) ASMR; (D, H) ASDR.

Supplementary file: Figure 6. SII analysis and Concentration index analysis. (A) The Interaction Effect of SDI Ranking and Gender on Incidence in 2021. (B) Concentrated Curves of Laryngeal Cancer Grouped by Gender and Year (1990 and 2021). (C) Concentration Curve of Cumulative Proportion and Incidence Rate of the Population Based on SDI Ranking. (D) Temporal Trend of SII from 1990 to 2021 (Divided by Male and Female Groups).

Supplementary file: Figure 7. Laryngeal cancer death rates attributable to tobacco, occupational risks and high alcohol use in 2021 globally, China and across SDI regions categorized by genders. (A) Proportion of laryngeal cancer mortality attributable to risk factors in the total population. (B) Proportion of laryngeal cancer mortality attributable to risk factors in male. (C) Proportion of laryngeal cancer mortality attributable to risk factors in female.

Supplementary file: Figure 8. Laryngeal cancer DALYs rates attributable to tobacco, occupational risks and high alcohol use in 2021 globally, China and across SDI regions categorized by genders. (A) Proportion of laryngeal cancer mortality attributable to risk factors in the total population. (B) Proportion of laryngeal cancer mortality attributable to risk factors in male. (C) Proportion of laryngeal cancer mortality attributable to risk factors in female.

Supplementary file: Figure 9. Trends in laryngeal cancer mortality attributable to risk factors in Global (A) and China (B) from 1990 to 2021.

Supplementary file: Table 1. Model fit indicators for INLA-BAPC projections (DIC and Shapiro-Wilk test p-values for residuals)

Supplementary file: Figure 10. Time trends of ASIR and ASMR in China and global LC from 1990 to 2050. blue dot lines and shaded regions represent the predicted trend and its 95% CI. (A) Predicted ASIR for China. (B) Predicted ASMR for China. (C) Predicted ASIR for the total population. (D) Predicted ASMR for the total population.

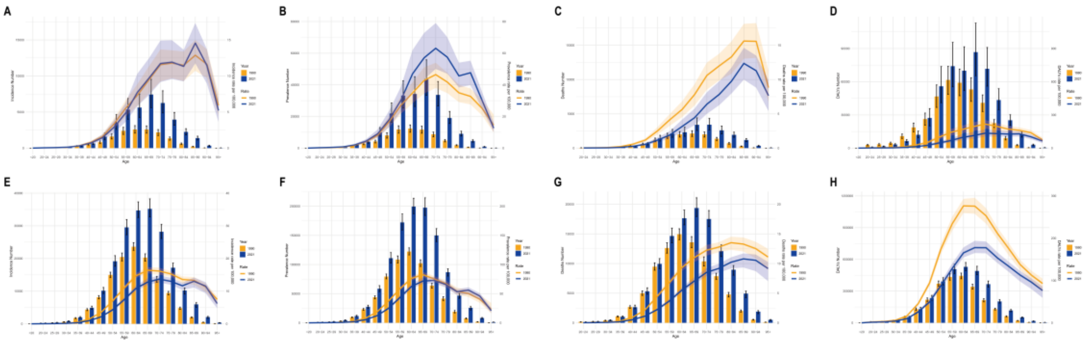

Supplementary file: Figure 1. Comparison of incidence rates, prevalence rates, mortality rates, and DALYs rates by age group and their crude rates from 1990 to 2021 (A–D: China; E–H: Globally). The error bars and shaded area represent the 95% UI. DALYs, disability-adjusted life years; UI, uncertainty interval.

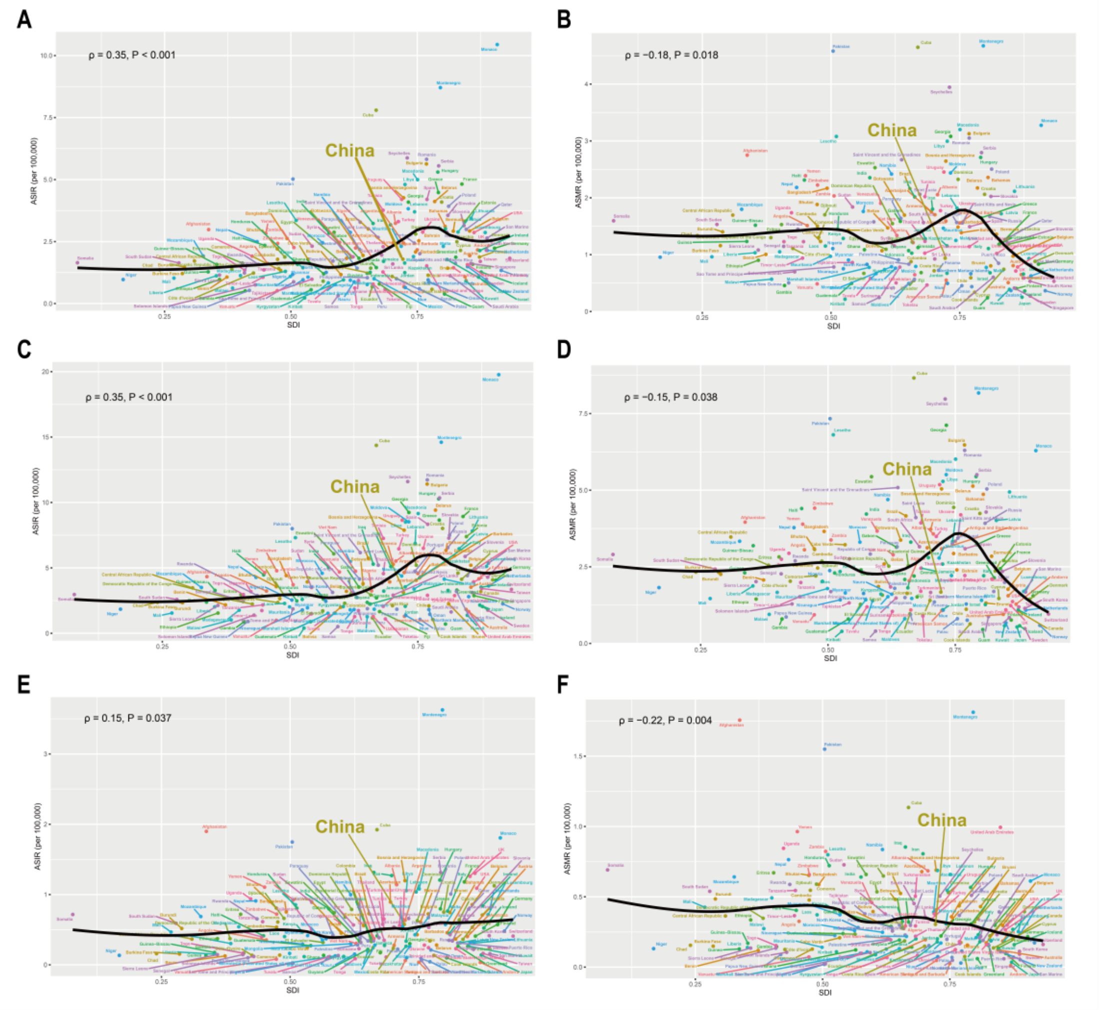

Supplementary file: Figure 2. Age-standardized burden rates of lung cancer across 204 countries and regions by Socio-demographic Index (SDI), 1990–2021. Each dot represents a country or region, and the burden rates include incidence (ASIR) and mortality (ASMR). The black line represents a smoothed trend fitted using LOESS regression across all observations. The ρ and p-values shown in each panel represent the Spearman correlation coefficient and its significance level between SDI and the respective burden rate. China is labeled for emphasis. (A–B) both sexes; (C–D) males; (E–F) females. ASIR: age-standardized incidence rate; ASMR: age-standardized mortality rate.

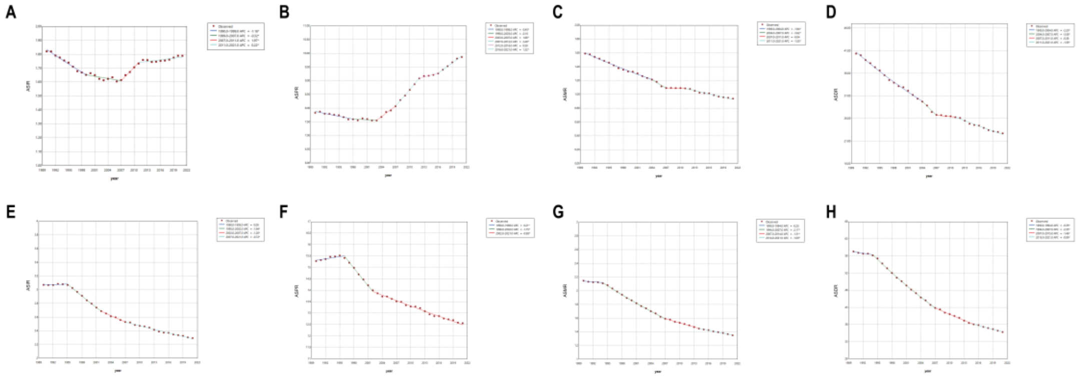

Supplementary file: Figure 3. The APC of ASIR, ASPR, ASMR, and ASDR in the total population of LC in China (A-D) and globally (E-H) from 1990 to 2021 (* indicates P-value < 0.05, statistically significant). (A, E) ASIR; (B, F) ASPR; (C, G) ASMR; (D, H) ASDR. APC, annual percent change**.** ASIR: age-standardized incidence rate; ASMR: age-standardized mortality rate. ASPR: age-standardized prevalence rate. ASDR: age-standardized DALYs rate. LC: laryngeal cancer.

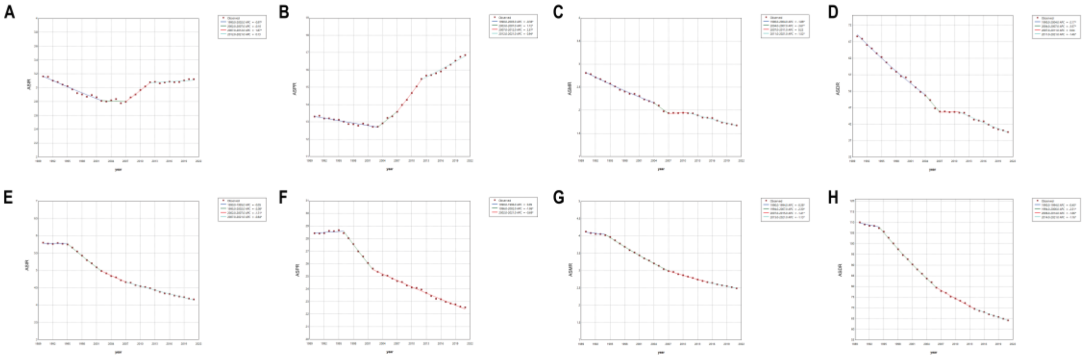

Supplementary file: Figure 4. The APC of ASIR, ASPR, ASMR, and ASDR in male LC in China (A-D) and globally (E-H) from 1990 to 2021 (* indicates P-value < 0.05, statistically significant). (A, E) ASIR; (B, F) ASPR; (C, G) ASMR; (D, H) ASDR**.** APC, annual percent change**.** ASIR: age-standardized incidence rate; ASMR: age-standardized mortality rate. ASPR: age-standardized prevalence rate. ASDR: age-standardized DALYs rate. LC: laryngeal cancer.

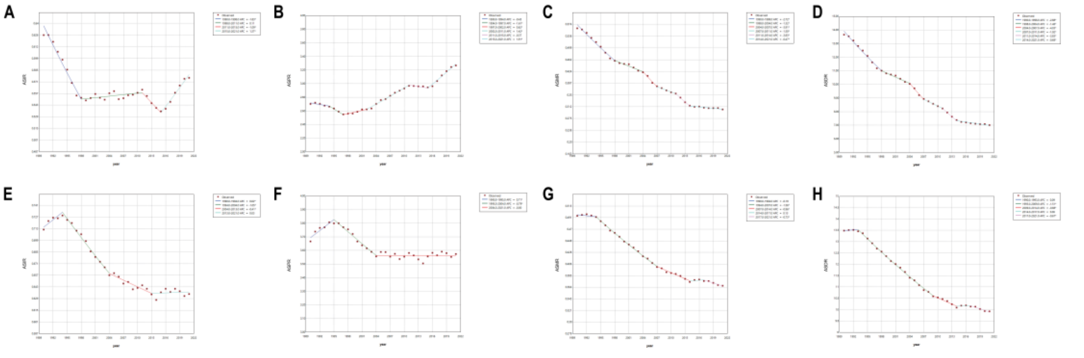

Supplementary file: Figure 5. The APC of ASIR, ASPR, ASMR, and ASDR in female LC in China (A-D) and

globally (E-H) from 1990 to 2021 (* indicates P-value < 0.05, statistically significant). (A, E) ASIR; (B, F) ASPR; (C, G) ASMR; (D, H) ASDR. APC, annual percent change. ASIR: age-standardized incidence rate; ASMR: age-standardized mortality rate. ASPR: age-standardized prevalence rate. ASDR: age-standardized DALYs rate. LC: laryngeal cancer.

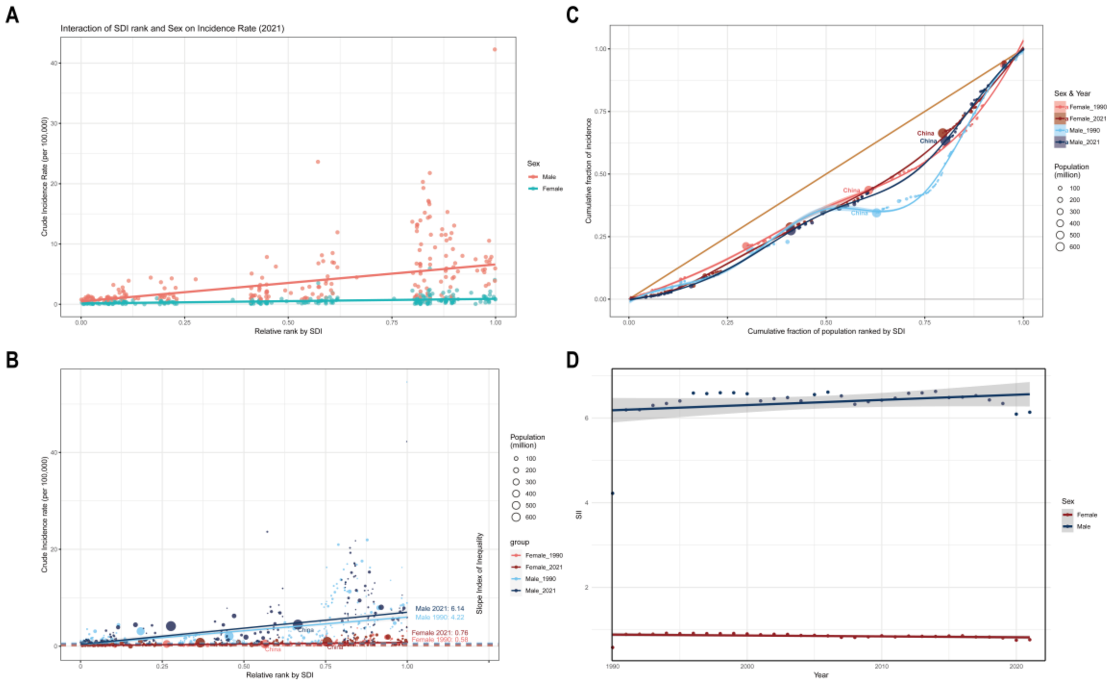

Supplementary file: Figure 6. SII analysis and Concentration index analysis. (A) The Interaction Effect of SDI Ranking and Gender on Incidence in 2021. (B) Concentrated Curves of Laryngeal Cancer incidence Grouped by Gender and Year (1990 and 2021). (C) Concentration Curve of Cumulative Proportion and Incidence Rate of the Population Based on SDI Ranking. (D) Temporal Trend of SII for incidence from 1990 to 2021 (Divided by Male and Female Groups). SII, Slope Index of Inequality; SDI, Socio-demographic Index

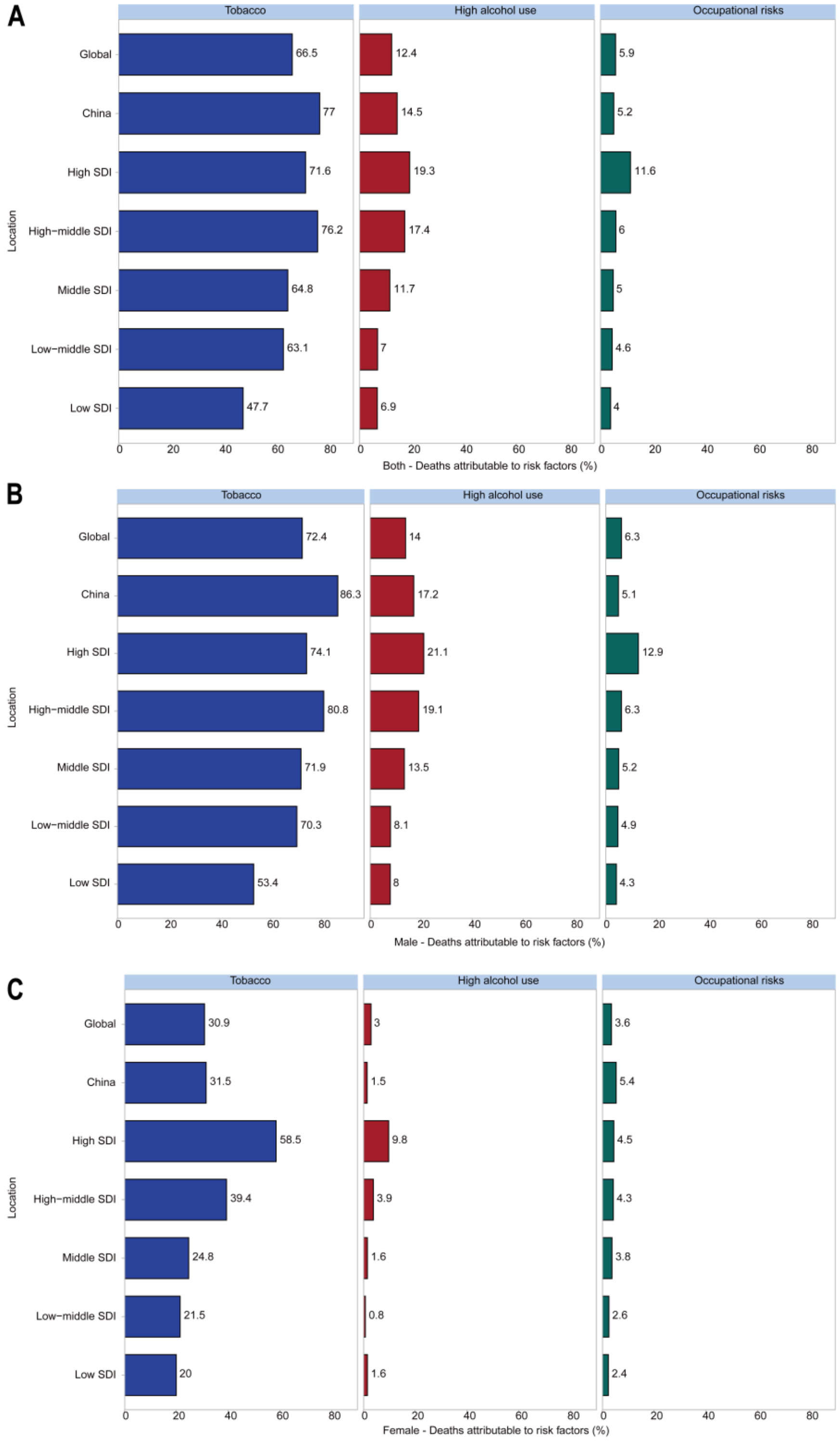

Supplementary file: Figure 7. Laryngeal cancer death rates attributable to tobacco, occupational risks and high alcohol use in 2021 globally, China and across SDI regions categorized by genders. (A) Proportion of laryngeal cancer mortality attributable to risk factors in the total population. (B) Proportion of laryngeal cancer mortality attributable to risk factors in male. (C) Proportion of laryngeal cancer mortality attributable to risk factors in female.

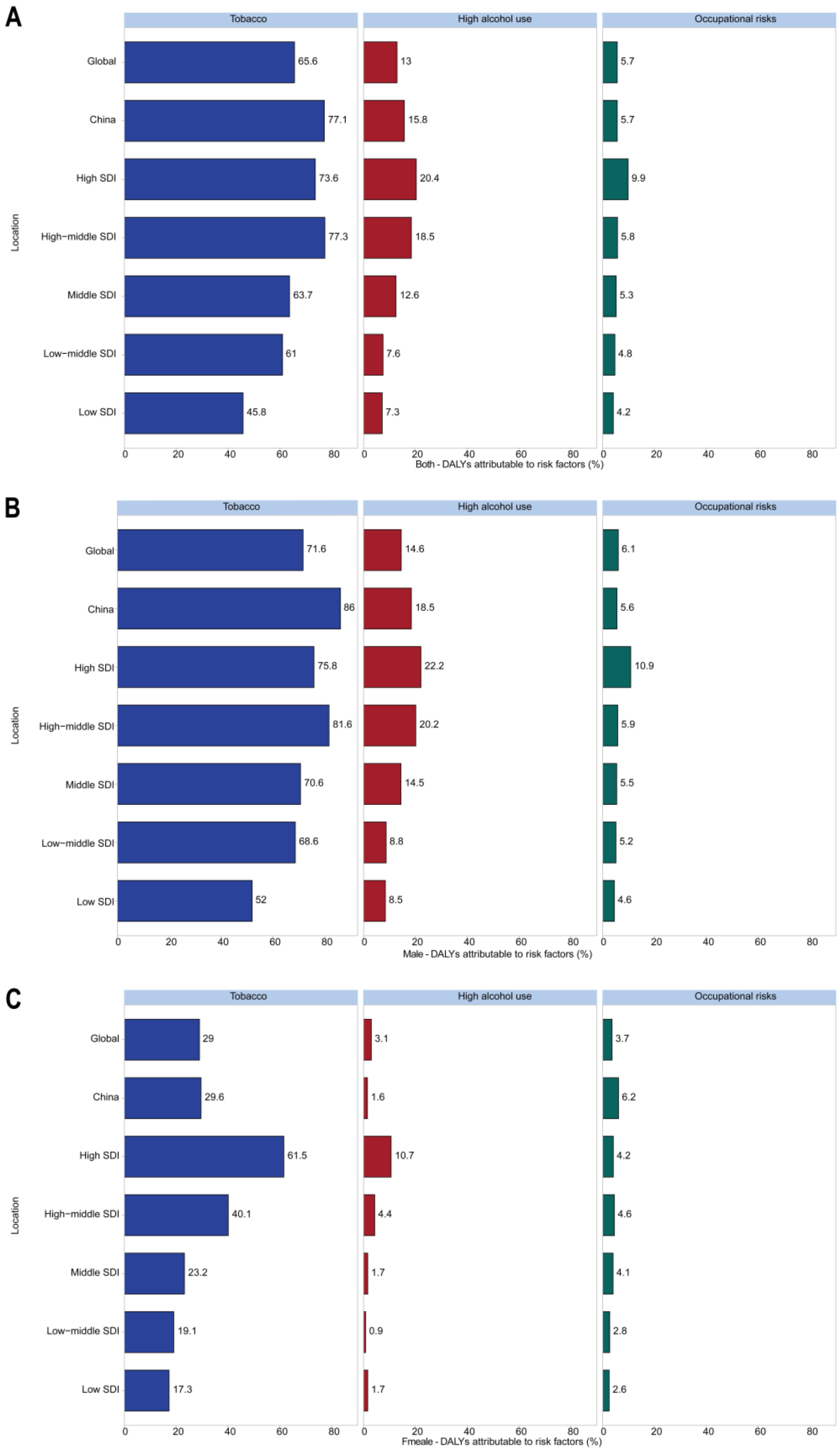

Supplementary file: Figure 8. Laryngeal cancer DALYs rates attributable to tobacco, occupational risks and high alcohol use in 2021 globally, China and across SDI regions categorized by genders. (A) Proportion of laryngeal cancer mortality attributable to risk factors in the total population. (B) Proportion of laryngeal cancer mortality

attributable to risk factors in male. (C) Proportion of laryngeal cancer mortality attributable to risk factors in female.

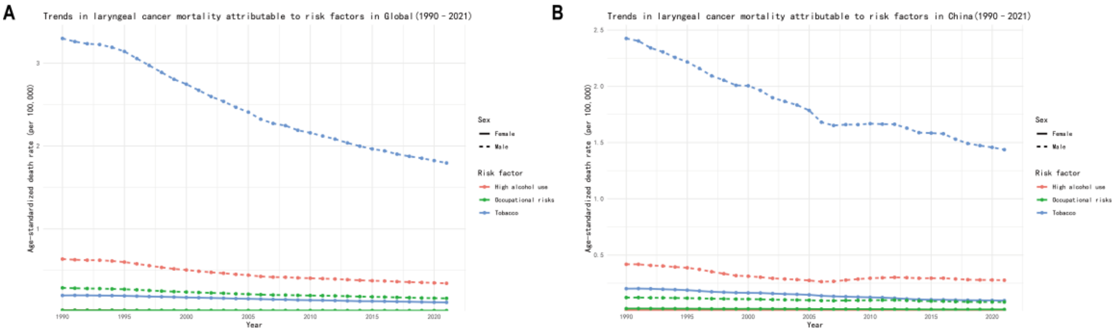

Supplementary file: Figure 9. Trends in laryngeal cancer mortality attributable to risk factors in Global (A) and China (B) from 1990 to 2021.

Supplementary file: Table 1. Model fit indicators for INLA-BAPC projections (DIC and Shapiro-Wilk test p-values for residuals)

Location	Measure	Sex	DIC	Shapiro-Wilk test (p-value)
China	Incidence	Both	4771.791	<0.05
		Male	4610.942	<0.05
		Female	3675.843	<0.05
	Deaths	Both	4549.557	<0.05
		Male	4375.493	<0.05
		Female	3441.294	<0.05
Global	Incidence	Both	5619.868	<0.05
		Male	5440.169	<0.05
		Female	4354.951	<0.05
	Deaths	Both	5911.122	<0.05
		Male	5735.617	<0.05
		Female	4603.435	<0.05

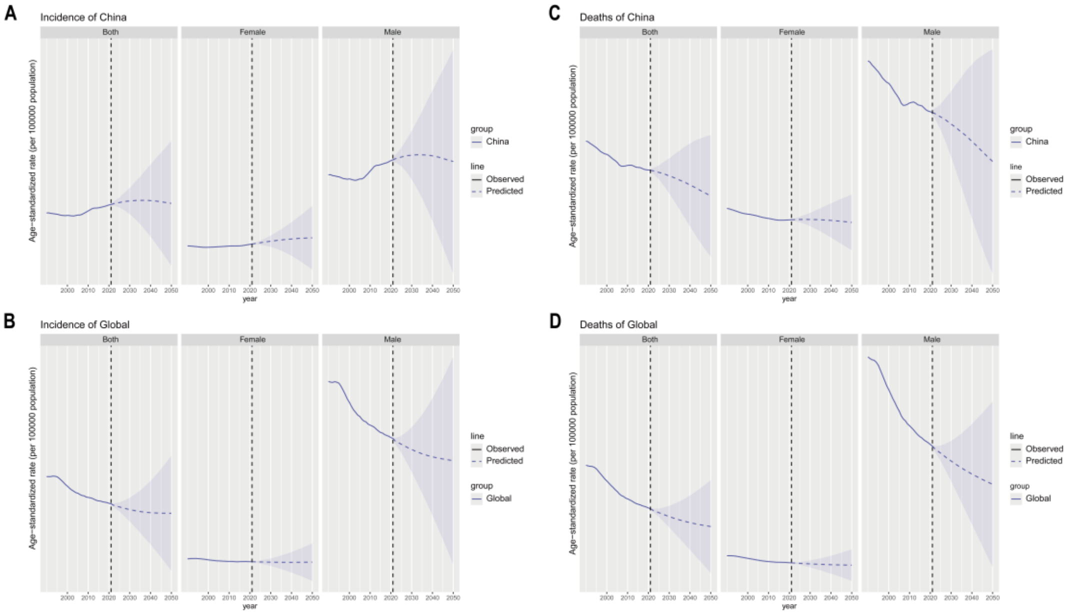

Supplementary file: Figure 10. Time trends of ASIR and ASMR in China and global LC from 1990 to 2050. blue dot lines and shaded regions represent the predicted trend and its 95% CI. (A) Predicted ASIR for China. (B) Predicted ASMR for China. (C) Predicted ASIR for the total population. (D) Predicted ASMR for the total population. APC, annual percent change**.** ASIR: age-standardized incidence rate; ASMR: age-standardized mortality rate. LC: laryngeal cancer.

© 2025 He Z. et al.
